# Modeling of the Lattice Dynamics in Strontium Titanate Films of Various Thicknesses: Raman Scattering Studies

**DOI:** 10.3390/ma16186207

**Published:** 2023-09-14

**Authors:** Veera Krasnenko, Alexander Platonenko, Aleksandr Liivand, Leonid L. Rusevich, Yuri A. Mastrikov, Guntars Zvejnieks, Maksim Sokolov, Eugene A. Kotomin

**Affiliations:** 1 Institute of Physics, University of Tartu, 50411 Tartu, Estonia; aleksandr.liivand@ut.ee; 2 Institute of Solid State Physics, University of Latvia, LV-1586 Riga, Latvia; a.platonenko@cfi.lu.lv (A.P.); leonid.rusevich@cfi.lu.lv (L.L.R.); yuri.mastrikov@cfi.lu.lv (Y.A.M.); guntars.zvejnieks@cfi.lu.lv (G.Z.); kotomin@latnet.lv (E.A.K.); 3 Theoretical Inorganic Chemistry, University of Duisburg-Essen, 45141 Essen, Germany; maksim.sokolov@uni-due.de; 4 Max Planck Institute for Solid State Research, 70569 Stuttgart, Germany

**Keywords:** STO, ultrathin films, Raman calculations, DFT, dependence on thickness

## Abstract

While the bulk strontium titanate (STO) crystal characteristics are relatively well known, ultrathin perovskites’ nanostructure, chemical composition, and crystallinity are quite complex and challenging to understand in detail. In our study, the DFT methods were used for modelling the Raman spectra of the STO bulk (space group I4/mcm) and 5–21-layer thin films (layer group p4/mbm) in tetragonal phase with different thicknesses ranging from ~0.8 to 3.9 nm. Our calculations revealed features in the Raman spectra of the films that were absent in the bulk spectra. Out of the seven Raman-active modes associated with bulk STO, the frequencies of five modes (2E_g,_ A_1g_, B_2g_, and B_1g_) decreased as the film thickness increased, while the low-frequency B_2g_ and higher-frequency E_g_ modes frequencies increased. The modes in the films exhibited vibrations with different amplitudes in the central or surface parts of the films compared to the bulk, resulting in frequency shifts. Some peaks related to bulk vibrations were too weak (compared to the new modes related to films) to distinguish in the Raman spectra. However, as the film thickness increased, the Raman modes approached the frequencies of the bulk, and their intensities became higher, making them more noticeable in the Raman spectrum. Our results could help to explain inconsistencies in the experimental data for thin STO films, providing insights into the behavior of Raman modes and their relationship with film thickness.

## 1. Introduction

Over the past decades, strontium titanate (STO) has established itself as an ideal research platform in various areas of solid-state physics and materials science, allowing the application of a variety of experimental and theoretical tools to study the physics and chemistry of perovskites ranging from defect chemistry, atomic-scale heterostructures and interfaces, polar hybrids and nanocomposites, lead-free nonlinear dielectrics and ferroelectric ceramics to phase transitions and the quantum effects of many-body physics [1,2,3,4,5,6,7,8,9,10,11,12,13,14,15,16]. SrTiO_3_ is a versatile perovskite structure material with a wide range of applications due to its unique properties. Moreover, the unique combination of polar, dielectric, and electronic properties in the perovskite structure has made strontium-titanate-based materials valuable for solid-state technologies such as catalysis, quantum electronics, and energy and electrical engineering. The technological applications of STO depend on its morphology, types of doping [17], and surface structure termination [18]. STO has shown promising results as a thin-film [19] and high-voltage capacitor [20], a bifunctional catalyst for CO_2_ hydrogenation [21], and a catalyst for water splitting reactions [15,22], superconductors [23], gas sensors [24,25], and substrate-based memory devices [26,27,28]. It is used as one of the main components of a crystalline host matrix for immobilization of high-level waste [29] obtained from spent fuel reprocessing in the nuclear industry and in perovskite-based solar cell applications [30,31].

The study of thin films is of great scientific and practical interest because thin-film systems can exhibit effects that are negligible or absent in bulk materials. In particular, the various structural defects that occur in thin films can dramatically affect their properties, whereas the presence of such defects is not as critical in bulk materials.

Stresses and strains are usually induced in thin films deposited on substrates [32,33]. Typically, for very thin films, the material tends to conform to the structural patterns of the substrate. However, when the film thickness is below the critical thickness, the thin film will accumulate elastic energy due to biaxial stresses caused by mismatched lattice parameters at the film–substrate interface. Because of this effect, the deformations remain distributed in the thin film even as it grows in size [34]. When the level of these strains begins to exceed the elastic limit of the material, stress relaxation processes occur, causing the film to become highly deformed. The relaxation processes are strongly dependent on the thickness of the film [33]. When the thickness of the thin film exceeds the critical [32] limit, edge dislocations usually form, causing a complete or partial elimination of the resulting deformations and stresses. In this case, the influence of the substrate is reduced, and the material acquires a spatial structure with a lattice constant equal or close to the lattice constant of the bulk under similar growth conditions. In this respect, a thin film of less than the critical thickness will have physical properties that are significantly different from those of thicker films [34].

Raman spectroscopy methods can be used to assess the structural and vibrational properties of materials of the same chemical composition but ordered in different dimensional configurations. Since Raman spectral peaks are given by characteristic values of the force constants in terms of scattering frequencies and spectral line intensities, Raman spectroscopy allows the analysis of differences in vibrational properties between a crystalline film and a bulk system. In general, the crystallinity of the film improves with increasing film thickness [35,36,37,38].

Compared to bulk samples, little progress has been made in studying the lattice dynamics of STO thin films using Raman spectroscopy techniques. Importantly, the Raman spectra measured for a number of STO thin-film samples have been found to be highly dependent on defects, film growth conditions, etc. For example, the Raman spectrum measured by Sirenko et al. [39] for three sample films (0.5, 0.75, and 2.3 μm thick) did not show a well-resolved peak that could be firmly associated with structural R modes (the zone-edge phonons becoming Raman active via double folding of the Brillouin zone due to structural phase transition from cubic to tetragonal). In contrast, measurements by Du et al. [40] in polycrystalline STO thin films (260 nm = 0.26 μm thick) allowed the identification of one of these modes through the detection of a Raman peak at 222 cm^−1^, which was attributed to the B_2g_ mode of the tetragonal phase. In previous studies of STO thin films grown on LaAlO_3_ substrates, first-order Raman scattering from transverse optical (TO) TO_1_ soft-mode phonons was not detected [39,41] but was observed in STO thin films grown on STO substrates with a SrRuO_3_ (SRO) buffer layer [42,43]. Banerjee et al. [44] observed a strong peak around 145 cm^−1^ in the experimental Raman spectrum of STO nanocubes, which was attributed to the Eg mode of the STO tetragonal phase. This feature was further investigated by Rabuffetti et al. [45], where it was found that the presence of such a peak strongly depends on the SrCO_3_ content in the sample. In addition, the results of the measurements and analysis by Gray et al. [46] showed that both Raman peaks detected at 145 and 448 cm^−1^ correspond only to the B_2g_ symmetry of the tetragonal phase. This is in contrast to previous reports [44,47,48,49,50] where the Raman peak at 145 cm^−1^ was attributed to nearly degenerate B_1g_ and E_g_ phonons [47,48] and the peak at 448 cm^−1^ to B_2g_ and E_g_ phonons [49,50].

The effect of film thickness on Raman spectral properties has also been investigated experimentally [39,41]. However, the results seem to be contradictory. On the one hand, the Raman studies of Akimov et al. [42] found that the soft-mode behavior in a 1 μm thick film became significantly harder than in the bulk structure. On the other hand, the measurements reported by Merkulov et al. [41] showed that the first-order Raman scattering peaks in STO films of different thicknesses (0.1, 0.35, and 1 μm) decreased by several cm^−1^ compared to their values in the bulk system. However, due to the strong effect of light scattering in thin-film samples, spectral features below 100 cm^−1^ were not examined in this work [41].

Such a lack of consistency in the results obtained from the experimental Raman spectra makes the problem of determining the actual dependence of the Raman activity on the thickness of STO thin films rather difficult. However, using quantum chemical approaches based on density functional theory (DFT), it is possible to simulate the theoretical Raman spectrum of STO films, which can be modeled for different film thicknesses. Unfortunately, very few theoretical works are reported to predict the Raman-active modes in ultrathin STO films, where the models containing only five [51] or six slabs [52] were considered. Since vibrational spectroscopy is an active field of research, we set out to fill in the missing amount of information needed to make spectroscopic protocols clear and understandable in the study of perovskite films. Thus, the following two challenges can be addressed. The first is to reproduce the Raman frequencies together with the symmetry characteristics of the Raman peaks in order to adequately describe the lattice dynamics of the bulk STO according to experiment. Second, spectral modeling can predict, in terms of systematic calculations of Raman spectral peaks, how the behavior of vibrational modes will depend on changes in the film thickness. Until now, this important issue related to the understanding of the peculiarities of the structure dynamics of thin films has not received much attention. Thus, the main goal of the present work is the theoretical reconstruction of the specific vibrational motions of atomic structural units, which may be active in the Raman spectrum of STO films. On the basis of calculations of Raman frequencies of the film, we intend to develop a model of the structure–spectral properties relationships, which could reflect the differences in the vibrational spectra between the film and bulk samples.

## 2. Materials and Methods

**Figure 1:**
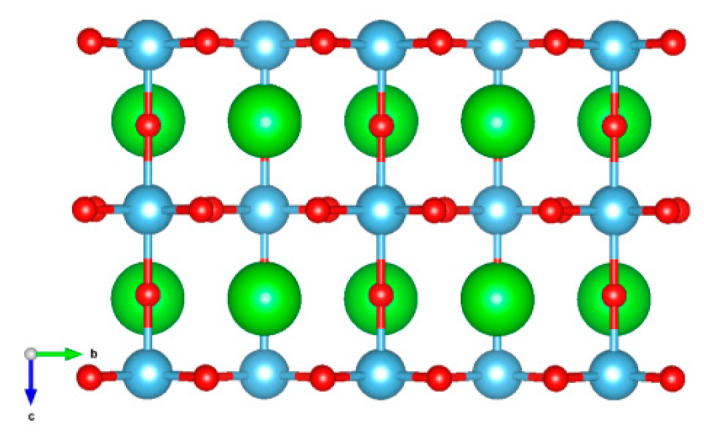
The five-layer TiO_2_-terminated (001) STO film with TiO_2_ central layer. Sr, Ti, and O atoms are the green, blue, and red spheres, respectively.

STO film was built from the bulk I4/mcm STO by creating a TiO_2_-terminated (001) slab with layer group p4/mbm (layer group No. 63) [51]. Thus, the bulk STO structure in the low-temperature tetragonal phase was taken as a matrix for the STO slabs. The structural layout of the STO film with five layers is illustrated in Figure 1. In order to evaluate the dependence of the vibrational properties on the film thickness when simulating Raman scattering, five different film models consisting of 5, 9, 13, 17, and 21 layers were considered. The chemical structure of the layers was represented by TiO_2_ and SrO compounds, and in all these cases, TiO_2_ served as the terminated (surface) and central layer.

The microstructural features of the models that we identified on the base of simulations are summarized in Table A1, Table A2 and Table A3 of Appendix A; they can be briefly outlined as follows:

1. The lattice parameters increase with the thickness of the STO films and tend to reach the values of the three-dimensional bulk structure (Table A1);
2. The surfaces of the film models appear uneven. Because there are no neighbors at the top, the atoms in the surface layers experience more uncompensated attraction from the atoms of the deeper layers, which, compared to the bulk model, leads to a shortening of the interatomic distances in the direction perpendicular to the surface (inward relaxation). As shown in Table A2, because the surface layers contain different atomic species, the chemical bonds between the surface and the second layer from the top are shortened differently, resulting in a surface-rumpling effect;
3. The dependence of the structural data on the film thickness calculated for the STO film models is given in Table A3. In agreement with the experimental data of Wang et al. [4], our results for the fully relaxed bonding geometries indicate that the in- and out-of-plane Sr–Sr distances increase and decrease, respectively, moving toward the values of the bulk STO. For film thicknesses between 1.5 and 3.9 nm (i.e., 9- and 11-layer films), no significant change in the out-of-plane lattice parameter near the film center was observed.

The first-principles calculations were performed within the linear combination of atomic orbitals (LCAO) approximation as implemented in CRYSTAL17 computer code [53]. Within the local density approximation (LDA) of DFT, the LDA exchange functional and VWN correlate functional [54] were used in calculations. (This combination of functionals is probably the most popular LDA formulation, also known as SVWN.) The basis set for strontium, titanium, and oxygen atoms was taken from a SrTiO_3_ study [55]. All basis sets are available online at CRYSTAL’s basis sets library [56]. The five threshold parameters governing the truncation of the Coulomb and exchange infinite lattice series were set to 9, 9, 9, 9, and 18. The integrations were performed on a default-predefined “extra extra large” pruned grid (XXLGRID) consisting of 99 radial points and 1454 angular points in the regions relevant for chemical bonding. A regular Monkhorst–Pack mesh of points in the reciprocal space was used for calculations with shrinking factor 8. The self-consistent field (SCF) convergence threshold parameter for the total energy was set to 10^−10^ Hartree ($2.7\times 10^{-9}\;\mathrm{eV}$) for both geometry optimization and vibration frequencies calculations. Raman intensities were evaluated through the couple-perturbed Hartree–Fock/Kohn–Sham (CPHF/CPKS) approach [57] as implemented in CRYSTAL to simulate the Raman spectra of the films studied. The quality of the optimizations performed reflects the fact that no imaginary modes were obtained in calculations of the vibrational properties.

One can also discuss the suitability of the functional LDA used in the present work for first-principles studies of lattice dynamics. It should be noted that the LDA tends to underestimate the experimental lattice constants by ~1%, which is clearly visible from the comparison of the first two rows of Table A1. This is a well-known fact that has been highlighted in previous studies of the structural dynamics of perovskites [58,59]. On the other hand, LDA gives good values for the tetragonality c/a [60]. It can be seen that the c/a ratio for the theoretical data of Table A1 remains within the experimental range for the bulk system. In order to minimize the effect of the LDA’s shortcoming, the following rules should be followed in the DFT calculations within the LDA: First, the modeling of vibrational properties should be based on theoretical values of the equilibrium lattice constants. This is due to the so-called sizeable error compensation that takes place when predicting LDA frequencies [61]. Other rules are dictated by (i) a careful choice of the basis and the scheme of the corresponding pseudopotentials [62,63] and (ii) the requirement to be able to take into account all factors that ensure a high level of accuracy of self-consistent calculations [64].

## 3. Main Results and Discussion

In Table 1, the symmetry properties of vibrational modes are summarized for both STO crystal and thin-film models. For instance, in accordance with other theoretical works (e.g., see [65,66]), the symmetry of phonon modes at the Γ point of the Brillouin zone in the I4/mcm tetragonal phase of the STO crystal can be characterized in terms of the allowed representations: eight IR-active modes, seven Raman-active modes, two acoustic A_2u_+E_u_ modes, and four silent modes.

Compared to the Raman activity of a crystal system, our simulations of STO film models revealed two key differences in vibrational properties: as indicated in Table 1, Table 2 and Table A4 (i), films can exhibit a greater number of Raman-active modes visible in the spectrum because new vibrational modes are induced due to surface effects, and (ii) the calculated Raman peaks indicate that the vibrational frequencies in the film differ from the values that are typical of the bulk structure. These results are in a good agreement with the experimental spectral data of Sirenko et al. [39], where the lowering of the crystal symmetry of STO films was observed through the appearance of strong optical phonon peaks that are forbidden in the Raman spectrum of the single crystal. An explanation for why spectral features are so strongly affected may be related to structural changes when a periodic system loses its bulk structure and acquires a lower-dimensional architecture. The scheme of change in atomic group displacements can be understood in terms of a corresponding change in symmetry. In particular, Table 1 shows that the geometry of 5-, 9-, 13-, 17-, and 21-layer films acquires the symmetry of the p4/mbm layer group.

**Figure 2:**
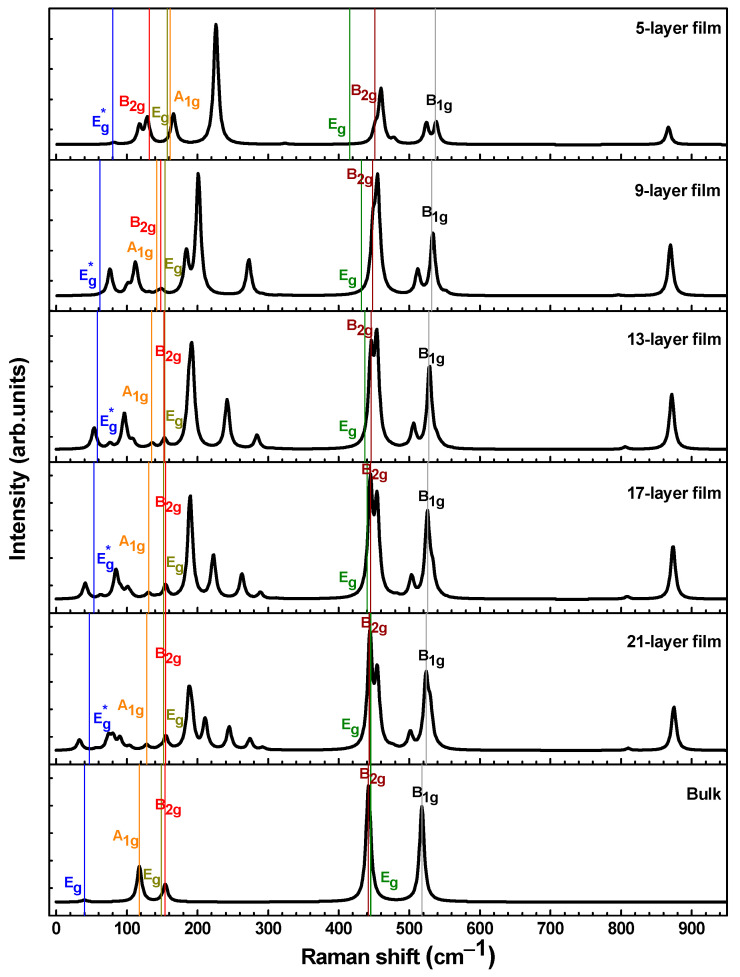
The theoretical Raman spectra calculated for the bulk and model of 5-, 9-, 13-, 17-, and 21-layer STO films. Modes with a superscript (asterisk) denote vibrations that correspond to a bulk mode only in the central layer (see Table 2 and Table A4 footnotes).

**Figure 3:**
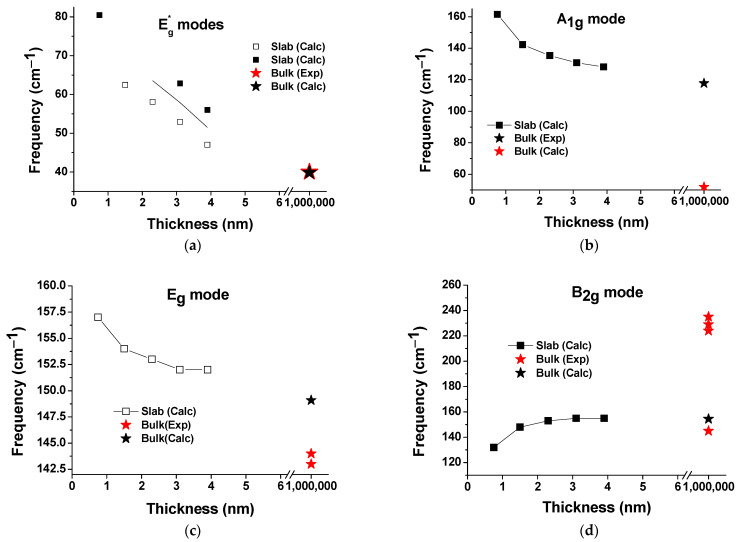

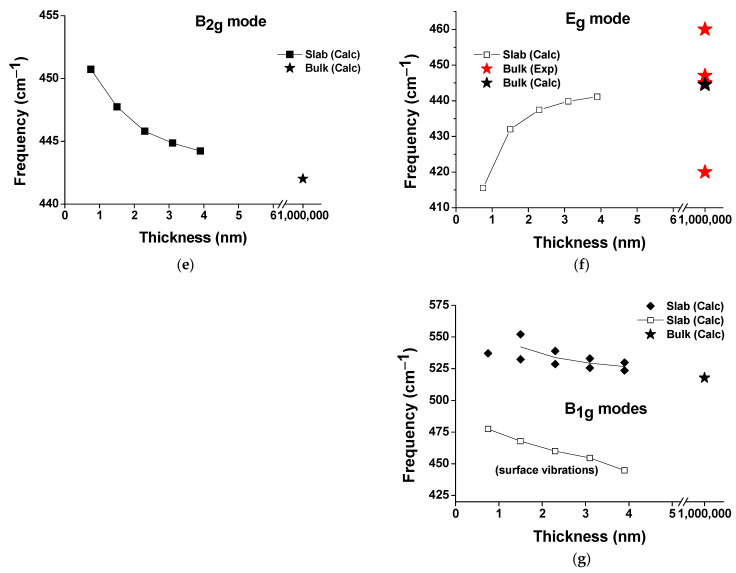
Experimental and calculated Raman-active modes (**a**–**g**) of bulk (red and black asterisks, respectively) vs. calculated similar modes in SrTiO_3_ films. The scatter of the experimental frequency values (red asterisks) is due to different experiments (see Table 2 and Table A4). The thickness of the bulk STO is given approximately. The calculated modes (**a**,**g**) correspond to different variants of the corresponding bulk STO mode. The modes with relative intensities near to zero are shown as empty squares. Modes with a superscript (asterisk) denote vibrations that correspond to a bulk mode only in the central layer (see Table 2 and Table A4 footnotes).

Figure 2 presents the theoretical Raman spectra calculated for the bulk and model 5-, 9-, 13-, 17-, and 21-layer STO films. The simulation results show that the changes in the internal structure of the STO film compared to the bulk structure are caused by local deformations due to a strong surface effect. That is, because of the significant role of finite-dimensional effects in the structure of the film, the constituent atoms can manifest themselves differently depending on their surroundings. This means that the spectral properties of vibrational modes, such as shape, amplitude, and frequency, can be directly affected. Another effect that we predicted from the simulations is the shifting of the frequencies of the Raman peaks active in the bulk structure (see Table 2 and Figure 3).

Depending on the thickness of the thin film, the values of the vibrational modes in the Raman spectra not only show a shift with respect to the bulk spectra (see Table 2) but are also characterized by different intensities of the Raman scattering peaks that occur as the scattering plane advances from the surface into the film thickness (see Figure 2). In addition, Raman scattering spectra may exhibit modes with such frequencies that are associated with a particular vibration typical of the bulk STO. At the same time, modes may appear that are simultaneously associated with both the STO films and the STO surface. These conclusions are consistent with the theoretical work of Blokhin et al. [51]. High-intensity modes in the range 850–900 cm^–1^ (only the A_1g_ modes are in this range) are associated with the STO surface since their vibrations in the central layer weaken with increasing film thickness, and starting from the thickness of the 13-layer film, vibrations in the central layers completely disappear (the thicker the film, the more central layers remain without vibrations—there are already 7 of them in the case of a 21-layer film). The surface mode B_1g_ (see Figure 3g—the bottom curve) was determined in the same way, which, however, due to its low intensity, is not visible in the Raman spectrum.

Moreover, although the vibrations of Ti atoms are only IR-active in bulk STO (see also [65,66]), in ultrathin STO films, these vibrations manifest themselves even in Raman-active modes (this is especially noticeable in E_g_ modes). According to our calculations, Ti atom vibrations are present in all vibrations of our models of ultrathin films except for B_2g_ and B_1g_ modes. In thin STO films, the Raman modes active in the bulk structure can have various vibrations characteristic and very different amplitudes in the central or surface parts of the films compared to bulk ones, which leads to shifts of these modes (see Figure 3a,g). To identify the STO bulk modes in our modelled films, we considered their specific patterns of vibrations (which atoms are involved in the vibrations of a particular mode and with what amplitudes) and intensities, focusing on the central as the most stable part of the films. According to our calculations, the frequencies of five out of the seven Raman-active modes characterizing bulk STO decrease with increasing thickness (see Figure 3a–c,e,g) except for the low-frequency B_2g_ and higher-frequency E_g_ modes, whose frequencies increase with increasing thickness (Figure 3d,f). As for the soft E_g_ mode, it mainly depends on the features of the vibrations of the film itself. In this case, the influence of the natural vibrations of the film is expressed in the vibrations of atoms that do not belong to the bulk mode E_g_ that vibrate with a larger amplitude than the atoms that belong to the mode E_g_ (so that only in the central layer the vibrations exactly correspond to this low-frequency mode E_g_). As expected, with increasing film thickness, the effect of film vibrations decreases. This explains the differences observed when comparing the Raman spectra of the bulk STO and films as well as the dependence of Raman spectra on the thickness effect. Moreover, the oxygen octahedra rotation angles of films differ from those in the bulk, also slightly varying from plane to plane, which is also reflected in extremely sensitive to local deviations from the average periodicity Raman spectra. Thus, simulation of the Raman spectra of ultrathin STO films yields results that differ from the spectra of bulk films, which is consistent with the experimental data [39,45,68].

The calculated dependence of the structural data of STO films on the film thickness is shown in Table A3. According to our data for fully relaxed films, the in-plane and out-of-plane Sr–Sr distances of the films were found to increase and decrease, respectively, approaching the values of bulk STO. Such behavior is in agreement with the experimental data of Wang et al. [35]. Starting from a film thickness of 2.3 to 3.9 nm (13-, 17-, and 21-layer films), no significant change in the out-of-plane lattice parameter near the center was observed.

Merkulov et al. [41] suggested that the observation of first-order Raman peaks in thin films is likely due to strain, which alters the crystal symmetry and makes detectable phonons previously inactive in Raman. Although stress and strain are always present in thin films deposited on substrates, our research suggests that the change in crystal symmetry is also a consequence of the finite system size effect. The surface–thickness relationship manifests itself in a preference for a surface where the environment of the atoms is different from that of the bulk. As the film thickness increases, its crystallinity improves, and the modes that characterize the crystal become more prominent in the Raman spectrum, as our calculations have shown.

**Figure 4:**
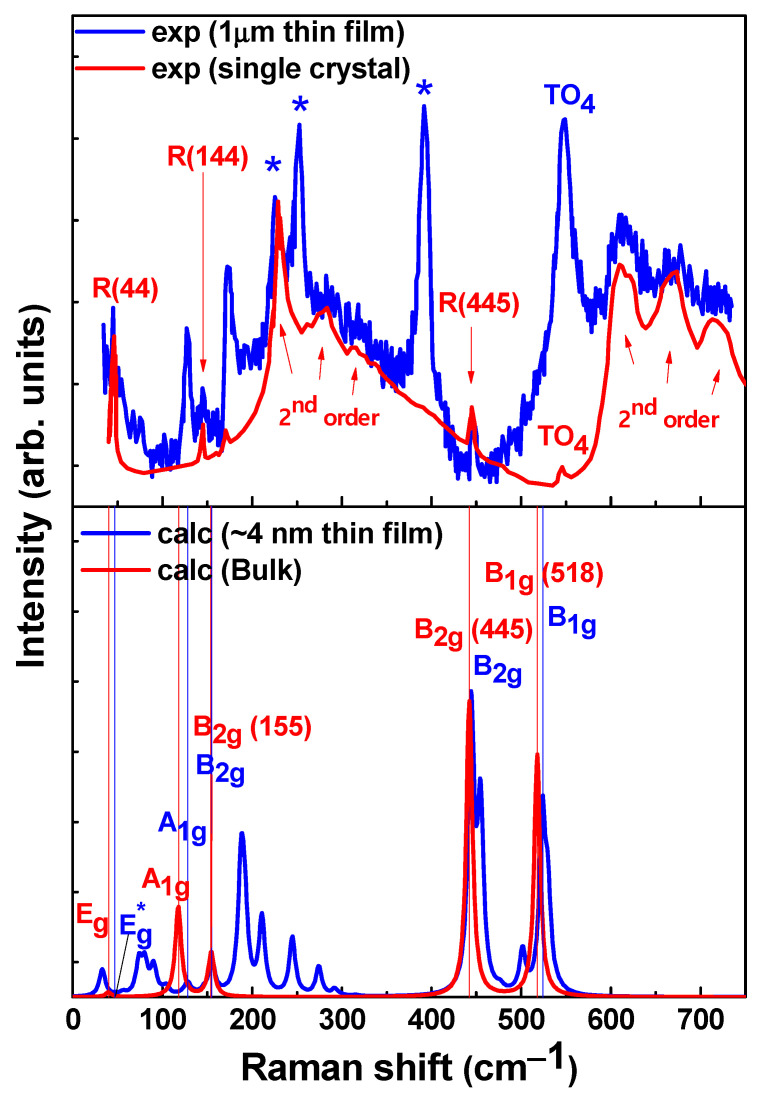
Raman shift of experimental tetragonal STO single crystal and 1 μm STO thin film (upper Figure) in the tetragonal phase [68] and calculated (lower Figure) bulk and 21-layer STO film (red and blue lines, respectively). Modes with a superscript (asterisk) denote vibrations that exactly correspond to a certain mode only in the central layer. Structural modes from experimental data in bulk STO are marked with R (cm^−1^). Optical phonons from the SrRuO_3_ buffer layer are marked with stars: STO thin films grown on STO substrates with a SrRuO_3_ buffer layer [39,68,72]. The experimentally observed first-order Raman scattering by hard-mode TO_4_ (Ti-O-Ti bending) phonons are marked with “TO_4_” [39,68]. The temperature of 10 K and the incident light of 514.5 nm (Ar + laser line for excitation) were used for experimental data.

According to our calculations (Figure 2 and Figure 4), the A_1g_, low-frequency B_2g_, and higher-frequency B_2g_ and B_1g_ modes, which relate to the bulk STO vibrations, are observed in the modelled Raman spectra. The frequencies of the low-frequency A_1g_ modes (rotation of the octahedra—rotation around tetragonal axis) of STO films decrease with increasing film thickness and become closer to those in bulk; some of these modes are not visible in the spectra due to very low intensities. The low-frequency B_2g_ modes increase, and their intensities become higher with increasing film thickness: starting from the 9-layer film, these modes became visible in the spectra. The higher-frequency B_2g_ and B_1g_ modes decrease with increasing film thickness: beginning from the 13-layer film, the peaks corresponding to these modes are clearly distinguishable in the Raman spectrum. B_2g_ modes are the most easily identified and stable; the values of these modes decrease only by several cm^−1^ with increasing film thickness (compared to their bulk values). The higher-frequency B_1g_ and lowest-frequency (soft) E_g_ modes undergo a thickness-dependent shifting with different intensities (see Table 2 and Table A4). As the film thickness increases, the wavenumber spread of the lowest-frequency E_g_ and B_1g_ modes becomes smaller and thus closer to the range of vibrational frequencies typical of the bulk STO.

Our calculations showed that the values of the higher-frequency (>400 cm^−1^) E_g_ modes of STO films related to the bulk increase with increasing film thickness; it seems that the lowest- and low-frequency E_g_ modes are the most sensitive to the film structure, as they are most affected by film vibrations. As expected, the closest values to the bulk values show the 21-layer film. Unfortunately, the Raman intensities of these E_g_ modes (including bulk ones) are extremely weak and are not visible in the calculated Raman spectra. This fact is in good agreement with the experimental observations of Gray et al. [46]. The lower film thickness also reduces the Raman intensity, causing some peaks of the single crystal to be too weak (compared to the new modes related to films; see Figure 2 and Figure 4 and Table A4) to distinguish in the Raman spectra. The same conclusion was reached by Merkulov et al. [41].

Considering that our films are extremely thin, our data are not in disagreement with the experiment, where only three structural R-modes—low-frequency structural soft mode (probably doublet A_1g_-E_g_, below 50 cm^−1^) and two higher-frequency R-modes (nearly degenerate E_g_ + B_2g_ phonons) [69,70]—are visible in the Raman spectra of a 1 μm thick SrTiO_3_ film (grown by pulsed laser deposition (PLD) on SrTiO_3_ substrates covered by 0.35 μm thick SrRuO_3_ buffer layers) [42,68]. According to Du et al. [40], only the B_2g_ mode appeared in the Raman spectra at 140 K and below, while the other R-point structural modes (e.g., at 48, 146, and 460 cm^−1^) were not detected in the polycrystalline STO thin film (with thickness 260 nm), which may also be caused by their lower intensities [40]. It should also be noted that in the bulk STO, Gray et al. [46] were only able to identify the soft modes E_g_-A_1g_ and two B_2g_ modes; i.e., only one (lowest-frequency) soft E_g_ mode was identified in their data, and no B_1g_ mode was identified. It should be also noted that the experimental data of Gray et al. [46] also differed in the interpretation of the peaks observed at 145 and 448 cm^−1^, where the value of 145 cm^−1^ for the B_2g_ mode is in agreement with our results [71] and most calculations of other authors (see e.g., [51,65,66,68]).

Even though the thicknesses of the structural models of our films are smaller than the experimental ones, we could be able to comment on why the peaks in the range of 180–250 cm^−1^ are present in the Raman spectra of thin STO film and absent in the spectrum of bulk STO. It was found that the peak around 190 cm^−1^ corresponds to A_1g_ and B_2g_ modes (not related to the bulk STO vibrations) that occur close to each other due to the shifting of corresponding modes in the thin films. This peak is related both to the vibrations of the STO surface and the film itself: the vibrations of the B_2g_ mode in the central layer weaken with increasing film thickness, and starting from the thickness of the 17-layer film, the vibrations in the central layers completely disappear. The vibrations of the A_1g_ mode are specific, including strong group (mostly all layers simultaneously) antiphase tensile-compression vibrations of Ti and Sr atoms along the Z-axis. Peaks around 210, 245, and 275 cm^–1^ (see Figure 4) correspond to the A_1g_ modes, which also differ from the corresponding mode of bulk STO and reflect the specifics of STO films as compared to the bulk: these modes include group (several layers simultaneously) antiphase tensile-compression vibrations of Ti and Sr atoms along the Z-axis.

When comparing the experimental data with the theoretical calculations of the Raman profiles, it could be observed that different substrates can cause some variations in the film thickness dependencies; for example, signal distortion due to inhomogeneous crystallinity, etc. This is mainly due to the mismatch in lattice characteristics, when the substrate structure can cause internal deformation or stress in the thin film. The occurrence of such an additional effect can affect the vibrational modes, which in turn leads to some shifts in the frequencies of the Raman peaks. On the other hand, the amount of strain can vary with film thickness, which also affects the Raman spectra. The substrate can also influence the chemical environment of the thin film by changing its symmetry and bonding properties as well as its surface roughness and morphology (i.e., surface roughness can scatter incident laser radiation differently, resulting in changes in Raman intensity). It is worth noting that some substrates can promote charge transfer or polarization effects in the thin film, which can also have an effect on the Raman spectra. In addition, one can mention the peculiarities of the spectral behavior of ultrathin films: when their thickness becomes of the order of (or less than) the mean phonon-free path, vibrational modes can be confined, resulting in a shift in the frequencies of the Raman peaks.

## 4. Conclusions

The calculated Raman spectra of the thin STO films have specific features that are not observed when evaluating spectra related to the bulk structure. This fact is in good agreement with the experimental data. We found that all Raman modes similar to those active in the bulk structure are present in the ultrathin STO films, although some of them are affected by film vibrations and are not visible in the Raman spectra due to extremely low intensities. Depending on the thin-film thickness, the vibrational modes in the Raman spectra are shifted with respect to the bulk ones, but in general, they show a tendency to eventually reach the values typical of bulk STO. Our simulations also show that the appearance of visible first-order Raman peaks in the spectra of thin films, which are absent in bulk STO Raman scattering, is related not only to substrate-related strains but also to changes in the structure of the film. The latter is caused by local structural strains due to the interplay of a stronger surface effect and the finite size of the system.

It is also worth noting that a large contribution to the experimental spectra in STO is made by the second-order Raman scattering reflecting the two-phonon density of states, which is a well-known spectral feature of this material (see e.g., [69,70]). Therefore, the simulation of the vibrational frequencies of the Raman (or IR) spectra is helpful in associating the vibrational modes appearing in the scattering spectrum with the vibrations of specific atoms, thus facilitating the interpretation of the experimental Raman and IR spectra.

Our models, which reproduce spectroscopic information for different lattice geometries based on density-functional calculations, implement a close interplay between theory and experimental data. This facilitates the calculation, visualization, interpretation, and reconstruction of vibrational spectra. It should be noted that the theoretical material presented in this paper will certainly be helpful in the analysis of vibrational spectra of multilayer (complex) films, which contain a variety of observable transitions from different ionic motions.

## Figures and Tables

**Table 1:** The group-symmetry-adapted modes for Γ point classified in terms of their spectral activity in infrared (IR) and Raman processes. The distribution of vibrations over irreducible representations is compared between the bulk structure (space group I4/mcm) and thin-film models (layer group p4/mbm).

STO	Raman-Active Modes	IR-Active Modes	Silent Modes
Bulk	1A_1g_ + 1B_1g_ + 2B_2g_ + 3E_g_	3A_2u_ + 5E_u_	2A_2g_ + A_1u_ + B_1u_
5-layer film	6A_1g_ + 2B_1g_ + 4B_2g_ + 10E_g_	6A_2u_ + 12E_u_	4A_2g_ + 4A_1u_ + 4B_1u_ + B_2u_
9-layer film	11A_1g_ + 3B_1g_ + 7B_2g_ + 19E_g_	11A_2u_ + 21E_u_	7A_2g_ + 7A_1u_ + 7B_1u_ + 2B_2u_
13-layer film	16A_1g_ + 4B_1g_ + 10B_2g_ + 28E_g_	17A_2u_ + 31E_u_	10A_2g_ + 10A_1u_ + 10B_1u_ + 3B_2u_
17-layer film	21A_1g_ + 5B_1g_ + 13B_2g_ + 37E_g_	22A_2u_ + 40E_u_	13A_2g_ + 13A_1u_ + 13B_1u_ + 4B_2u_
21-layer film	26A_1g_ + 6B_1g_ + 16B_2g_ + 46E_g_	27A_2u_ + 49E_u_	16A_2g_ + 16A_1u_ + 16B_1u_ + 5B_2u_

**Table 2:** Comparison of experimentally observed Raman-active phonon modes of single-crystal STO with those (similar or corresponding) obtained from our calculations for ultrathin film (layer group p4/mbm) and the bulk (space group I4/mcm) models.

Modes	Frequency (cm^−1^)						
	5 Layers	9 Layers	13 Layers	17 Layers	21 Layers	Bulk (Calc)	Bulk (Exp)
$E_{g}^{*}\;$	80.46 ^(1)^	62.46 ^(1)^	58.08 ^(1)^	52.93 ^(1),(2)^ 62.83 ^(1),(3)^	47.03 ^(1),(2)^ 55.99 ^(1),(3)^	39.85	11 [67], 15 [47], 40 [48]
A_1g_	161.47	142.23	135.35	130.79	128.14	117.74	44 [68], 48 [47], 52 [48]
E_g_	157.29	153.80	153.03	151.61	152.02	149.06	143 [47], 144 [68]
B_2g_	132.36 ^(3)^	148.17 ^(3)^	153.29 ^(3)^	154.78 ^(3)^	155.18 ^(3)^	154.48	145 [46], 224 [45], 229 [48], 235 [47]
B_2g_	450.73 ^(3)^	447.76 ^(3)^	445.81 ^(3)^	444.86 ^(3)^	444.22 ^(3)^	442.02	
E_g_	415.56 ^(3)^	432.02 ^(3)^	437.48 ^(3)^	439.85 ^(3)^	441.13 ^(3)^	444.51	420 [67], 445 [68], 447 [48], 460 [47]
B_1g_	477.37 ^(2)^	461.42 ^(2)^	452.9 ^(2)^	447.98 ^(2),(4)^	444.82 ^(2),(4)^	517.80	
	537.11 ^(3)^	532.37 ^(3)^	528.57 ^(3)^	525.62 ^(3)^	523.57 ^(3)^		
		552.17 ^(3)^	538.94 ^(3)^	533.00 ^(3)^	529.80 ^(3)^		

^(1)^ Only in the central layer do the vibrations exactly correspond to this mode (such modes are denoted with *, e.g., $E_{g}^{*}$); ^(2)^ vibrations of surface atoms with higher amplitudes than vibrations in the central layer; ^(3)^ vibrations in the central layer with higher amplitudes than vibrations of surface atoms; ^(4)^ there are no vibrations in the central layer (and adjacent to the central layers).

## Appendix A

**Table A1:** The space groups and the calculated lattice parameters of the bulk (space group I4/mcm) and the 5-, 9-, 13-, 17-, and 21-layer STO films (layer group p4/mbm).

STO	Lattice Parameters					
	a (Å)	b (Å)	c (Å)	Alpha (°)	Beta (°)	Gamma (°)
Bulk exp ^1^	5.510	5.510	7.798	90	90	90
Bulk	5.455	5.455	7.755	90	90	90
5-layer film	5.393	5.393	-	90.00	90.00	90.00
9-layer film	5.418	5.418	-	90.00	90.00	90.00
13-layer film	5.429	5.429	-	90.00	90.00	90.00
17-layer film	5.434	5.422	-	90.00	90.00	90.00
21-layer film	5.438	5.438	-	90.00	90.00	90.00

^1^ Landolt-Börnstein. Numerical data and functional relationships in science and technology. New Series. Group III., Vol. 36A1 (Springer-Verlag, 2001); [62].

**Table A2:** Calculated (LDA) thickness of TiO_2_-terminated STO films. The different values of the thicknesses are due to displacements of atoms along the normal to the surfaces.

STO Film	Thickness (Å)	Atom
5-layer	7.60	Ti
	7.76	O
9-layer	15.36	Ti
	15.52	O
13-layer	23.12	Ti
	23.28	O
17-layer	30.88	Ti
	31.03	O
21-layer	38.63	Ti
	38.79	O

**Table A3:** The calculated structural data of STO films with different thicknesses and STO crystal. The different values of the thicknesses are due to displacements of atoms along the normal to the surfaces.

STO	Thick-Ness, Å	Sr–Sr between SrO Layers, Å	Sr–Sr in SrO Layers, Å	Ti–Ti between TiO_2_ Layers (Out-Of-Plane), Å	Ti–Ti in TiO_2_ (In-Plane), Å	Ti–O in TiO_2_ Layers, Å	O–O between TiO_2_ Layers, Å
5-layer film	7.60 (Ti–Ti) 7.76 (O–O)	4.11	3.81	3.80	3.81	Central layer 1.92, surface layer 1.91	3.89
9-layer film	15.36 (Ti–Ti) 15.52 (O–O)	Near to center 3.92, near to surface 4.00	3.83	Near to center 3.88, near to surface 3.80	3.83	Central layer and between 1.93, surface layer 1.92	Near to center 3.9, near to surface 3.89
13-layer film	23.12 (Ti–Ti) 23.28 (O–O)	Near to center (2 SrO layers) 3.89, between 3.91, near to surface 3.99	3.84	Near to center 3.89, between 3.87, near to surface 3.80	3.84	1.93, surface layer 1.92	3.91, near to surface 3.86
17-layer film	30.88 (Ti–Ti) 31.03 (O–O)	Near to center (4 SrO layers) 3.89, between 3.90, near to surface 3.99	3.84	Near to center 3.89, between 3.88, near to surface 3.87, surface 3.80	3.84	1.93, surface layer 1.92	3.91, surface layer 3.86
21-layer flm	38.63 (Ti–Ti) 38.79 (O–O)	Near to center (6 SrO layers) 3.89, between 3.90, near to surface 3.98	3.85	Near to center 3.89, between 3.88, near to surface 3.87, surface 3.79	3.85	1.93, surface layer 1.92	3.90, surface layer 3.86
Bulk		3.87	3.86	3.88	3.86	1.94	3.88

**Table A4:** The experimental Raman-active modes of bulk STO (pure crystal) and calculated frequencies and relative intensities (in parentheses) of phonon modes (corresponding to similar bulk modes) in ultrathin films with layer group p4/mbm.

Modes	Frequency, cm^−1^ (Intensity, Arb. Units)						
	5 Layers	9 Layers	13 Layers	17 Layers	21 Layers	Bulk (Calc)	Bulk (Exp)
$E_{g}^{*}$	80.46 ^(1)^ (16.32)	62.46 ^(1)^ (4.36)	58.08 ^(1)^ (0.98)	52.93 ^(1),(2)^ (0.29) 62.83 ^(1),(3)^ (26.86)	47.03 ^(1),(2)^ (2.8) 55.99 ^(1),(3)^ (11.6)	39.85 16.63)	11 [67], 15 [47], 40 [48]
A_1g_	161.47 (4.46)	142.23 (19.69)	135.35 (33.32)	130.79 (38.44)	128.14 (39.49)	117.74 (323.56)	44 [68], 48 [47], 52 [48]
E_g_	157.29 (1.13)	153.80 (1.76)	153.03 (0.58)	151.64 (0.99)	152.02 (0.84)	149.06 (5.53)	143 [47], 144 [68]
B_2g_	132.36 ^(3)^ (2.88)	148.17 ^(3)^ (40.55)	153.29 ^(3)^ (94.75)	154.78 ^(3)^ (115.06)	155.18 ^(3)^ (118.34)	154.48 (157.28)	145 [46], 224 [45], 229 [48], 235 [47]
B_2g_	450.73 ^(3)^ (109.74)	447.76 ^(3)^ (471.86)	445.81 ^(3)^ (880.15)	444.86 ^(3)^ (1000)	444.22 ^(3)^ (1000)	442.02 (1000)	
E_g_	415.56 ^(3)^ (0.22)	432.02 ^(3)^ (0.82)	437.48 ^(3)^ (1.49)	439.85 ^(3)^ (1.69)	441.13 ^(3)^ (1.71)	444.51 (81.5)	420 [67], 445 [68], 447 [48], 460 [47]
B_1g_	477.37 ^(2)^ (33.77)	461.42 ^(2)^ (23.69)	452.91 ^(2)^ (23.99)	447.98 ^(2),(4)^ (18.83)	444.82 ^(2),(4)^ (14.42)	517.80 (868.17)	
	537.11 ^(3)^ (177.18)	532.37 ^(3)^ (515.84)	528.57 ^(3)^ (814.40)	525.62 ^(3)^ (759.07)	523.57 ^(3)^ (617.54)		
		552.17 ^(3)^ (1.96)	538.94 ^(3)^ (61.10)	533.00 ^(3)^ (197.07)	529.80 ^(3)^ (318.38)		

^(1)^ Only in the central layer do the vibrations exactly correspond to this mode (further such modes are denoted with *, e.g., $E_{g}^{*}$); ^(2)^ vibrations of surface atoms with higher amplitudes than vibrations in the central layer; ^(3)^ vibrations in the central layer with higher amplitudes than vibrations of surface atoms; ^(4)^ there are no vibrations in the central layer (and adjacent to the central layers).
